# Multiple spacer sequence typing of *Coxiella burnetii* carried by ticks in Gansu, China

**DOI:** 10.3389/fvets.2024.1470242

**Published:** 2024-11-27

**Authors:** Ze-Yun Xu, Fang-Ni Wang, Rui Jian, Jing Xue, Ya-Chun Guo, Wen-Ping Guo

**Affiliations:** College of Basic Medicine, Chengde Medical University, Chengde, Hebei, China

**Keywords:** ticks, *Coxiella burnetii*, *IS1111*, 16S rRNA, genotyping, MST

## Abstract

**Background:**

*Coxiella burnetii* is a zoonotic pathogen that causes Q fever and is found worldwide. Ticks serve as the primary reservoir, playing an important role in maintaining the natural cycle of *C. burnetii*. *C. burnetii* is transmitted to animals when ticks feed on their blood. However, information on *C. burnetii* infection in ticks remains limited, despite the widespread prevalence of the infection in humans and animals across China.

**Methods:**

In this study, 192 engorged ticks were collected from Baiyin City of Gansu Province, China. The presence of *Coxiella burnetii* in ticks was specifically identified by detecting the *IS1111* gene using nested polymerase chain reaction (nPCR). In addition, the 16S rRNA gene of *C. burnetii* was molecularly characterized using nPCR. A total of 10 spacer sequences (Cox 2, 5, 18, 20, 22, 37, 51, 56, 57, and 61) were amplified using PCR against positive specimens for MST analysis.

**Results:**

All collected ticks were identified as *Hyalomma marginatum*, and 90 of them tested positive for *C. burnetii*, with a positive rate of 46.9% (90/192). The 16S rRNA gene analysis showed that the novel *C. burnetii* variants detected in this study were closely related to other *C. burnetii* strains in the world. The allele codes found in the present study for loci Cox2-Cox5-Cox18-Cox20-Cox22-Cox37-Cox51-Cox56-Cox57-Cox61 were 8-4-9-5-7-5-2-3-11-6. This represents a novel combination of allele values, similar to MST28, currently designated as MST85 in the Multi Spacers Typing (MST) database.

**Conclusion:**

Our results revealed the circulation of a novel MST genotype of *C. burnetii* in Baiyin City, Gansu Province, China. The detection of *C. burnetii* in ticks suggests a potential public health risk to the local human population.

## Introduction

Q fever, caused by the obligate intracellular bacterium *Coxiella burnetii*, is a worldwide disease that can infect both animals and humans (1). It was first reported in Australia in 1935 (2), with the Netherlands having the highest prevalence (3). Q fever has spread to almost all countries worldwide (4). It is now one of the most widely distributed zoonotic diseases, affecting the health of both humans and animals (5, 6). Humans become infected mainly from animals through infected aerosols and the ingestion of raw milk or dairy products (6). Human infection can manifest with chills, fever, and headache (6, 7). At the same time, severe cases of Q fever presenting complications such as hepatitis, endocarditis, rare spinal infections, prosthetic joint infections, and even death have been reported (6–9). Meanwhile, since Q fever is not a legally reported infectious disease in China, its clinical symptoms are atypical and not emphasized and thus difficult to diagnose (10). The rate of clinical misdiagnosis and underdiagnosis is high, and the disease is also easily neglected (11).

Ticks can transmit *C. burnetii* to animals while feeding on their blood, and these animals can subsequently transmit the agent to humans (12). In addition, *C. burnetii* direct transmission to humans by ticks through biting has been reported, though it is rare (13); hence, its potential risk to humans should be considered. To date, more than 40 hard ticks from genera *Haemaphysalis, Amblyomma, Rhipicephalus, Hyalomma*, and *Dermacentor* and at least 14 soft ticks from *Ornithodoros* have been documented as vectors for *C. burnetii* (14–17). In China, *C. burnetii* has been identified in *Hyalomma* (18–20), *Dermacentor* (19–22), *Rhipicephalus* (19, 23), and *Haemaphysalis* (19–22, 24).

Multi Spacers Typing (MST), utilized for genotyping *C. Burnetii* (5, 25), not only reflects the predominant genotypes in each region but also enables strain sequence typing comparisons, thereby facilitating traceability to the source of *C. burnetii* infection (5, 25, 26). MST can be directly applied to DNA extracted from specimens without the need to culture pathogen isolates, offering the advantage of high reproducibility between laboratories (27). To date, 79 MST types have been identified based on the MST database, and only MST16 has been found in rats from Yunnan, China (28). Furthermore, four potential novel MST types were identified, including two in rats from Yunnan (28) and another two in hedgehogs from Hubei (29).

Baiyin City is located in the central part of Gansu Province, near Lanzhou, and at least two species of ticks have been identified within its territory. Although *C. burnetii* has been identified in *D. nuttalli, D. silvarum, Ha. japonica*, and *Hy. asiaticum* from Gansu Province (20, 22), no studies on *C. burnetii* infection in ticks have been reported in Baiyin City, Gansu. In the present study, *C. burnetii* was screened in ticks from Baiyin City, and the MST types of *C. burnetii* were identified to determine its prevalence in ticks within the region.

## Materials and methods

### Collection and identification of ticks and DNA extraction

**Figure 1:**
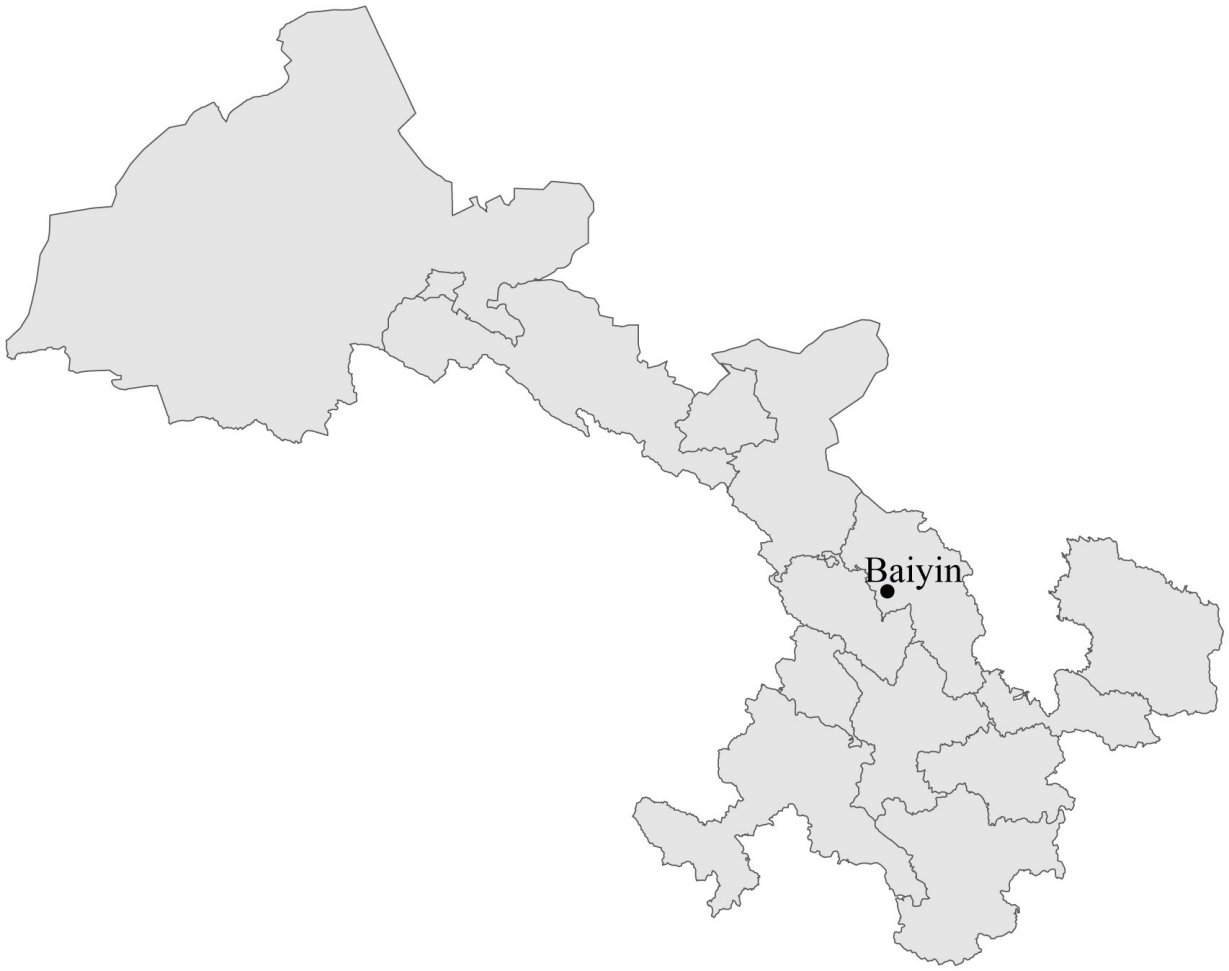
Map with the location of the collection site of ticks (•) in Baiyin City, Gansu Province, China.

In August 2019, one adult tick was collected from the body of each goat in Baiyin City, Gansu Province (Figure 1). Ticks were initially identified at the species level based on morphological characteristics under a stereoscopic microscope. The taxonomical keys, mainly including the shape of the basis capitulum, palp, scutum, coxae I, anal groove, eyes, festoons, adanal plates, spiracle, and hypostomal teeth, were used for the identification of tick species. In addition, tick species were confirmed by analyzing the cytochrome c oxidase I (*COI*) gene sequence obtained using polymerase chain reaction (PCR) (30). All collected tick specimens were washed twice with 75% alcohol and then washed twice with phosphate-buffered saline (PBS). Following the manufacturer's instructions, total DNA was extracted from each tick using the tissue DNA extraction kit (Omega, Norcross, GA, USA). The extracted DNA sample was eluted into 80 μl ddH_2_O and stored at −80°C before screening *C. burnetii*.

### Molecular identification and characterization of *C. burnetii*

*Coxiella burnetii* was screened by amplifying the *IS1111* gene using nested polymerase chain reaction (nPCR). Primer pair QBT1/QBT2 was used for the first round of nPCR (31), and QBTN3/QBTN4 was used for the second round of nPCR (32), yielding a 440-bp amplicon.

To better understand the genetic characteristics, a partial 16S rRNA gene (624–627 bp) was amplified from the samples positive for *C. burnetii* using nPCR. Primer pairs Cox16S-F1/16S-R and 16S-F/16S-R were used as the first and second rounds, respectively (33). All primers used in this study are shown in Table 1.

**Table 1:** Primer sequences used in this study.

**Target gene**	**Primer**	**Oligonucleotide sequences (5′- 3′)**	**Amplicon size (bp)**	**References**
IS1111	QBT1	TATGTATCCACCGTAGCCAGTC	687	(31)
	QBT2	CCCAACAACACCTCCTTATTC		
	QBTN3	AAGCGTGTGGAGGAGCGAACC	440	(32)
	QBTN4	CTCGTAATCACCAATCGCTTCGTC		
16S rRNA	16S-F1	CGTAGGAATCTACCTTRTAGWGG	624-627	(33)
	16S-F	TGAGAACTAGCTGTTGGRRAGT		
	16S-R	GCCTACCCGCTTCTGGTACAATT		
COX2	COX2F	CAACCCTGAATACCCAAGGA	397	(34)
	COX2R	GAAGCTTCTGATAGGCGGGA		
COX5	COX5F	CAGGAGCAAGCTTGAATGCG	395	
	COX5R	TGGTATGACAACCCGTCATG		
COX18	COX18F	CGCAGACGAATTAGCCAATC	557	
	COX18R	TTCGATGATCCGATGGCCTT		
COX20	COX20F	GATATTTATCAGCGTCAAAGCAA	631	
	COX20R	TCTATTATTGCAATGCAAGTGG		
COX22	COX22F	GGGAATAAGAGAGTTAGCTCA	383	
	COX22R	CGCAAATTTCGGCACAGACC		
COX37	COX37F	GGCTTGTCTGGTGTAACTGT	463	
	COX37R	ATTCCGGGACCTTCGTTAAC		
COX51	COX51F	TAACGCCCGAGAGCTCAGAA	674	
	COX51R	GCGAGAACCGAATTGCTATC		
COX56	COX56F	CCAAGCTCTCTGTGCCCAAT	479	
	COX56R	ATGCGCCAGAAACGCATAGG		
COX57	COX57F	TGGAAATGGAAGGCGGATTC	617	
	COX57R	GGTGGAAGGCGTAAGCCTTT		
COX61	COX61F	GAAGATAGAGCGGCAAGGAT	611	
	COX61R	GGGATTTCAACTTCCGATAGA		

### MST genotype of *C. burnetii*

MST was performed to determine the genotypes of *C. burnetii* using PCR to target 10 spacers with the highest variability, as previously described (34). These spacers include Cox2, Cox5, Cox18, Cox20, Cox22, Cox37, Cox51, Cox56, Cox57, and Cox61. All primers used in this study are shown in Table 1.

### Sequencing and nucleotide sequence analysis

The PCR products were analyzed using electrophoresis on a 1% agarose gel, and the spacer sequence PCR products were analyzed on a 1.2% agarose gel. All the PCR products of the expected size were purified and cloned into pMD19-T vectors (Takara, Dalian, China) for sequencing with the universal primers (Sangon, Beijing, China).

Bioedit v. 7. 1. 11 was used to edit all newly generated sequences in this study (35). The obtained *IS1111* and 16S rRNA genes were analyzed using BLAST comparison on the NCBI website. The nucleotide sequence identities were calculated using the MegAlign program available within the Lasergene software package (36). To better understand the relationship between the *C. burnetii* identified in this study and other strains, the maximum-likelihood (ML) tree was reconstructed based on the 16S rRNA gene sequence using MEGA 6.0.6 software (37). The optimal nucleotide substitution model General Time Reversible (GTR) nucleotide substitution model as well as the gamma (G)-distribution and proportion of invariable sites (i.e., GTR+G+ I) were determined using the MEGA 6.0.6 (37). Bootstrap values were calculated from 1,000 replicates, and the phylogenetic trees were rooted at the midpoint for clarity.

Individual spacer sequences of *C. burnetii* obtained in this study were concatenated. The MST genotype was determined by comparing the results with the MST database of *C. burnetii* (https://ifr48.timone.Univ-mrs.Fr/mst/coxiella_burnetii/ accessed on 24 April 2024). A phylogenetic tree of the MST genotypes was built using the unweighted pair group method with the arithmetic mean method (UPGMA) using MEGA 6.0.6 (37). A minimum spanning tree was generated using the software GrapeTree with parameters implemented in MSTree v2 (http://localhost:8000/) for the 10 alleles from all STs (38).

## Results

### Identification of *C. burnetii* and ticks

**Figure 2:**
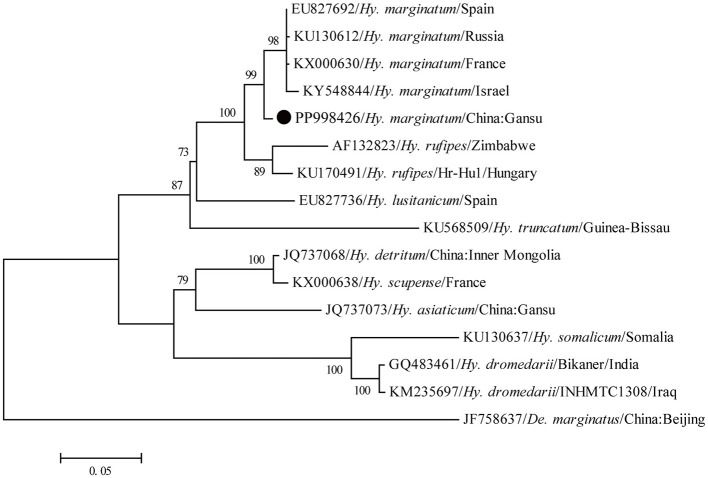
Molecular identification of ticks based on the phylogenetic analysis with the *COI* gene. The maximum-likelihood (ML) tree was reconstructed using the MEGA 6.0.6 under the GTR+G+I model with 1,000 replicates. The numbers at each node indicated bootstrap values, and only bootstrap values >70% are shown at appropriate nodes. Taxa marked by circles depict representative sequences obtained in this study.

A total of 192 ticks were collected from the body of goats, and all ticks were identified as *Hy. marginatum* based on the morphology. Subsequently, the *COI* gene sequence obtained from all ticks showed 99.4–100% nucleotide identity with each other and exhibited 97.7–98.7% nucleotide identity with known sequences of this tick species deposited in the GenBank database (GenBank numbers: OQ799122, PP330223, and KX000648). Furthermore, in the phylogenetic tree based on the *COI* gene, all newly generated sequences in this study had the closest relationship with those of *Hy. marginatum* (Figure 2). Therefore, all these ticks were confirmed to be *Hy. marginatum*.

Gel electrophoresis analysis showed that the size of the 90 PCR products was in accordance with the expected size. Sequencing of the PCR products and further BLAST showed that all these newly generated sequences most closely resembled those of *C. burnetii* and shared the highest nucleotide identity of 97.2–100% nucleotide identity with known *IS1111* gene sequences of *C. burnetii*. The positive rate of *C. burnetii* infection in *Hy. marginatum* ticks was 46.9% (90/192). Moreover, all these 90 *IS1111* gene sequences presented 99.5–100% nucleotide identity with each other. All the *IS1111* gene sequences obtained in this study have been submitted to GenBank under the accession numbers PP929917–PP930006.

### Molecular characterization of 16s rRNA gene of *C. burnetii*

To better understand the genetic characteristic, a partial 16S rRNA gene was successfully amplified from 56 out of 90 *C. burnetii*-positive tick specimens. After sequencing, 56 partial 16S rRNA gene sequences showed 99.1–100% nucleotide identity with known those of *C. burnetii* from the GenBank database. Furthermore, all these 56 partial 16S rRNA gene sequences presented 99.8–100% nucleotide identity with each other. All the 16S rRNA gene sequences obtained in this study have been submitted to the GenBank database under the accession numbers PP930513–PP930568.

**Figure 3:**
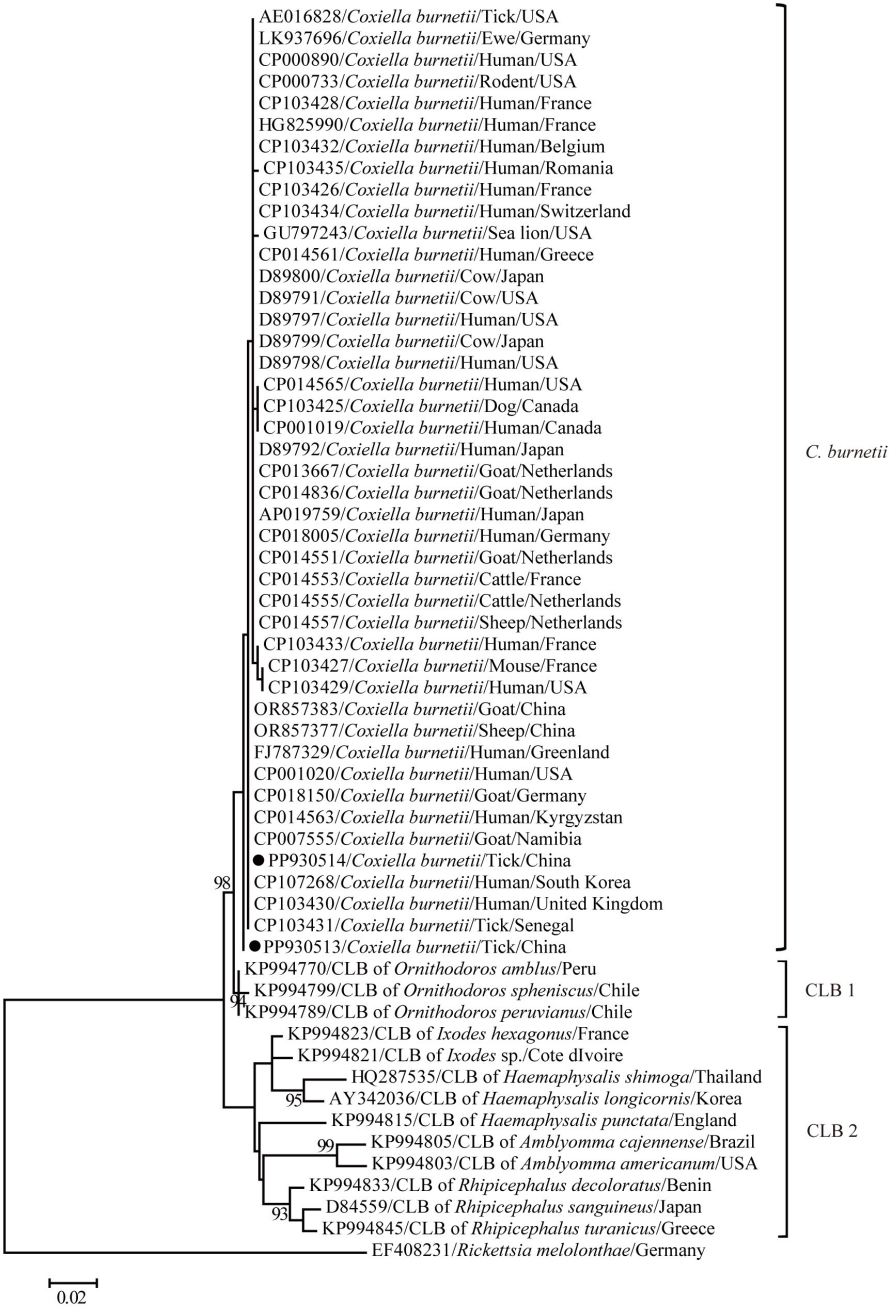
A phylogenetic tree based on the 16S rRNA gene. The numbers at each node indicate bootstrap values, and only bootstrap values >70% are shown at appropriate nodes. Taxa marked by circles depict representative sequences obtained in this study.

The maximum-likelihood tree based on the partial 16S rRNA gene sequences was reconstructed to get a better understanding of the relationships between the *C. burnetii* variants determined in this study and other known strains. In general, clear segregation into three clusters was observed in the partial 16S rRNA gene tree in this study: *C. burnetii*, CLB1, and CLB2 (Figure 3). *Coxiella burnetii* variants identified in this study clustered together with other known *C. burnetii* strains including those identified from humans and separated from two groups of *Coxiella* endosymbiont (CLB) (Figure 3). Furthermore, *C. burnetii* was closely related to CLB1 and distantly related to CLB2. Consistently, *C. burnetii* shared 98.4–99.3% and 93.8–97.7% nucleotide identities with CLB1 and CLB2 for the partial 16S rRNA gene, respectively. In addition, CLB1 was distantly related to CLB2 and only shared 93.3–96.9% nucleotide identity.

### MST genotyping of *C. burnetii*

**Figure 4:**
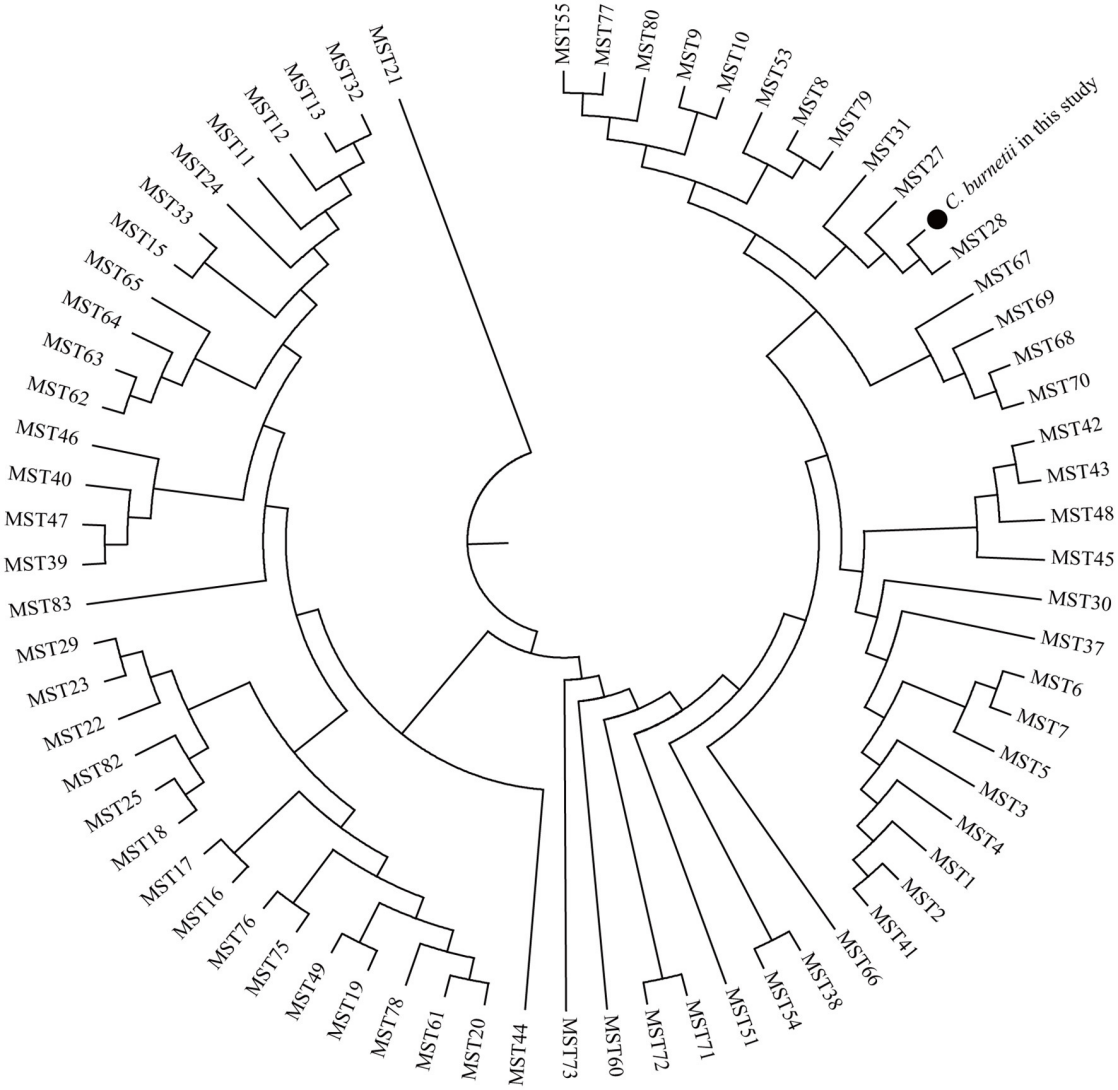
Phylogenetic tree of *C. burnetii* MST genotypes identified in this study with known genotypes. The MST genotype identified in this study and known MST genotypes from the MST database (https://ifr48.timone.univ-mrs.fr/mst/coxiella_burnetii/) were used. Phylogenetic analysis was performed using the unweighted pair group method with the arithmetic mean (UPGMA) method. Taxa marked by circles depict the sequences obtained in this study.

**Figure 5:**
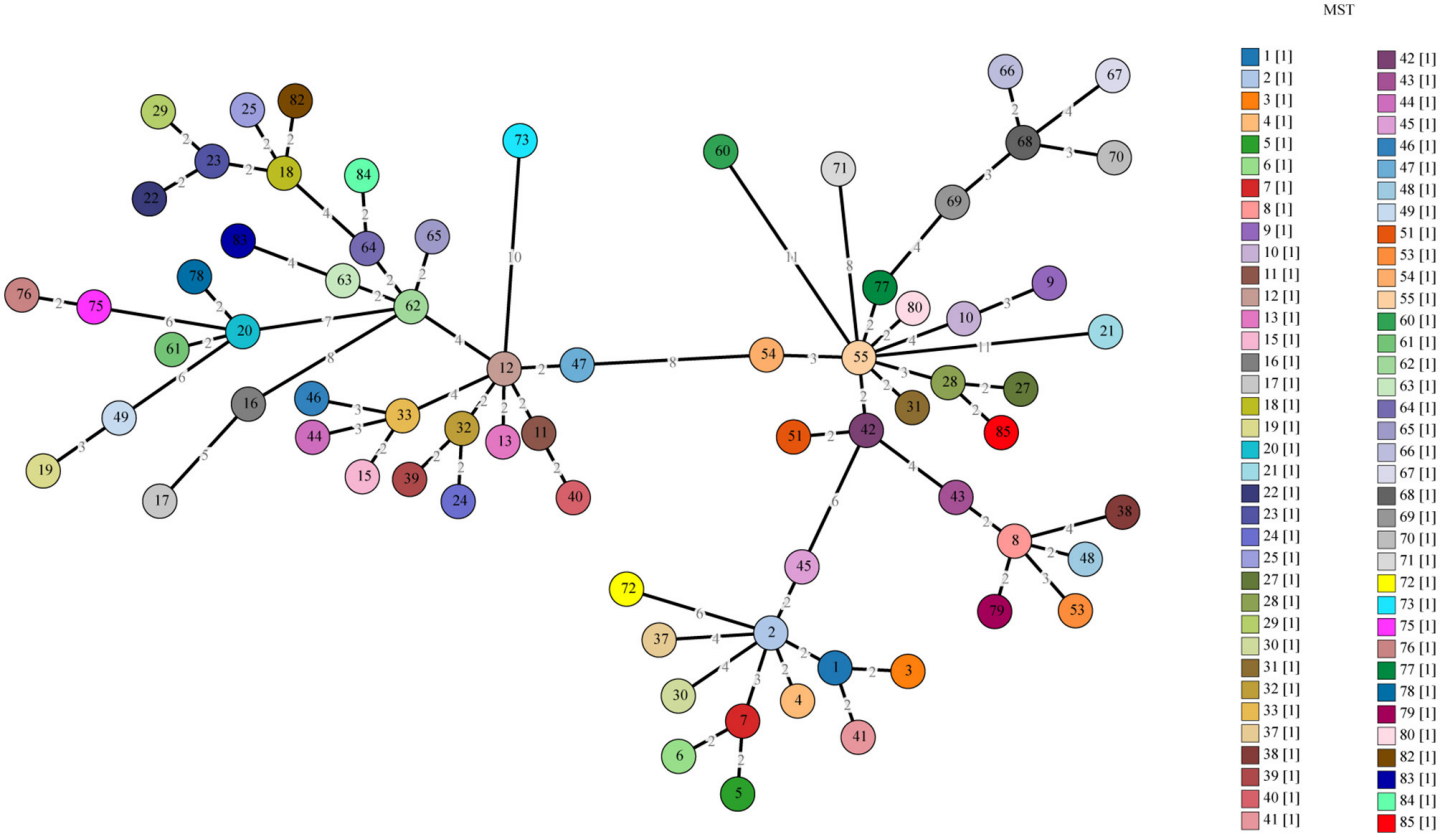
A minimum spanning tree for the ten allele profiles with all STs.

Allelic loci were successfully obtained at 10 allelic intervals from 55 tick samples (Table 2). The allele codes found in the present study for loci Cox2-Cox5-Cox18-Cox20-Cox22-Cox37-Cox51-Cox56-Cox57-Cox61 were 8-4-9-5-7-5-2-3-11-6. The allele values of the single spacer sequences of *C. burnetii* in different specimens are shown in Table 2. The 10 successfully amplified spacer sequences were combined and compared to the sequences in the MST database, and the results showed that the allele values identified in this study were a novel combination of allele values similar to MST28 found in sheep, cattle, ticks, and humans from Kazakhstan, Central Asia (Figure 4). Compared to MST28, which has an allele value of 4 for the Cox57 spacer, this combination of allele values showed a value of 11, which has been found in MST types 66–70, indicating a unique MST genotype. This novel MST genotype has been submitted to the MST database, currently defined in the database as MST85. The minimum spanning tree constructed from 10 alleles of all STs showed that MST28 was a putative ancestral genotype for MST27 and MST85 (Figure 5).

**Table 2:** *Coxiella burnetii* genotyping based on multiple spacer sequence typing.

**Sample category**	**Sample number**	**Intergenic spacer**										**MST genotype**
		**COX2**	**COX5**	**COX18**	**COX20**	**COX22**	**COX37**	**COX51**	**COX56**	**COX57**	**COX61**	
1	55	8	4	9	5	7	5	2	3	11	6	Novel
2	2	8	4	9	5	7	5	2	3	NA	6	NI
3	1	8	4	9	5	7	5	2	NA	11	6	NI
4	4	8	4	9	NA	7	5	2	3	11	6	NI
5	7	8	4	NA	5	7	5	2	3	11	6	NI
6	1	8	4	NA	5	7	5	2	NA	11	6	NI
7	1	8	4	NA	5	7	5	2	3	11	NA	NI
8	2	8	4	NA	NA	7	5	2	3	11	6	NI
9	1	8	NA	9	5	7	5	2	3	11	6	NI
10	1	8	NA	9	NA	NA	5	2	NA	NA	6	NI
11	1	8	NA	9	NA	NA	NA	2	3	11	6	NI
12	1	8	NA	NA	5	7	5	2	3	NA	6	NI
13	1	8	NA	NA	NA	7	5	2	3	11	6	NI
14	1	8	NA	NA	NA	NA	NA	2	NA	11	NA	NI
15	1	8	NA	NA	5	7	5	2	3	11	6	NI
16	4	NA	4	9	5	7	5	2	3	11	6	NI
17	1	NA	4	9	5	7	NA	NA	3	NA	6	NI
18	1	NA	4	9	NA	7	5	2	3	11	6	NI
19	1	NA	4	9	NA	NA	5	NA	3	NA	NA	NI
20	2	NA	NA	9	5	7	5	2	3	11	6	NI
21	1	NA	NA	NA	5	NA	NA	2	NA	NA	NA	NI

NA, not amplified; NI, not identified.

## Discussion

Q fever has always been a public health problem of concern in the international community (4, 39). Worldwide, Q fever epidemics have occurred in recent years in some countries, including Chile (40), Ethiopia (41), Iran (42), and the Netherlands (43). Q fever was first reported in China in 1950, and the first isolation of *C. burnetii* was performed in 1962 from a patient with chronic Q fever (44). In Chinese history, small outbreaks of Q fever have occurred in Xizang, Xinjiang, and Inner Mongolia, and sporadic cases of Q fever have been reported in 64 cities/municipalities across 19 provinces (19, 44). Although direct transmission from ticks to humans is scarce, *C. burnetii* infection in ticks can reflect its threat to local domestic animals and further reflect its risk to local populations. However, there is insufficient information on tick-borne *C. burnetii* in China. Therefore, a better understanding of the epidemiology of *C. burnetii* infection in ticks would be helpful for the prevention and control of Q fever in humans. In this study, *C. burnetii* was identified in *Hy. marginatum* ticks from Baiyin City of Gansu Province, China. This finding is consistent with previous reports of *C. burnetii* in several species within the genus *Hyalomma*. The positive rate of *C. burnetii* infection in *Hy. marginatum* ticks in this study was 46.9%, which was higher than that in *D. Nuttalli, Hy. asiaticum, D. silvarum*, and *Ha.japonica* collected from other areas of Gansu Province (20, 22). The different positive rates may be related to the detection method, collection site, and ecological environment.

It is well-known that rRNA operons are weakly affected by horizontal gene transfer (45–47), and no recombination occurred in these regions (48). Thus, the 16S rRNA gene plays a crucial role in species identification and construction of phylogenetic relationships of prokaryotes, including *Coxiella*. The 16S rRNA gene sequences obtained in this study had the highest homology and clustered with other known *C. burnetii* sequences, suggesting that the pathogen detected in this study should be considered as *C. burnetii*. The 16S rRNA gene obtained in this study had 100% nucleotide identity and presented a close genetic relationship with *C. burnetii* variants from humans including Ammassalik (FJ787329) (49), CbuK_Q154 (CP107268) (50), and Schperling (CP014563) (51), suggesting a high risk of its infection in the local population.

MST is a well-established genotyping method for *C. burnetii*, which is of great significance for the traceability of geographical and natural host sources for Q fever (52). Currently, 80 *C. burnetii* MST genotypes worldwide are stored in the MST database. In recent years, the application of MST genotyping technology for *C. burnetii* has also been reported in China. MST16 was found in wild rats from Yunnan (28), and two potential novel MSTs were identified in hedgehogs from Hubei Province (29). However, MST genotyping of *C. burnetii* identified in ticks has yet to be reported in China. In this study, a novel MST was identified and characterized by a novel combination of known allele values. This novel MST was identified from the majority of *C. burnetii*-positive tick samples, suggesting that it is the predominant MST genotype in Baiyin City.

The main limitation of this study is that we cannot rule out the possibility that *C. burnetii* may have originated from the blood meal, as the ticks were collected from goats. Therefore, it is still unclear whether *Hy. marginatum* ticks can serve as the effective vector of *C. burnetii*. However, only *Hy. marginatum* ticks were collected in the endemic areas of *C. burnetii* in this study; therefore, *Hy. marginatum* may be the vector of *C. burnetii* in the local area, which should be confirmed in future studies.

## Conclusion

In this study, *C. burnetii* was found in *Hy. Marginatum* in Gansu Province, China. A novel MST defined as MST85, similar to MST28, was identified. It is necessary to study the *C. burnetii* carried by ticks to provide a theoretical basis for the prevention and control of Q fever in China.

## Data Availability

The data presented in the study are deposited in the GenBank repository, accession number PP929917-PP930006 and PP930513-PP930568.
